# External validation of four dementia prediction models for use in the general community-dwelling population: a comparative analysis from the Rotterdam Study

**DOI:** 10.1007/s10654-018-0403-y

**Published:** 2018-05-08

**Authors:** Silvan Licher, Pınar Yilmaz, Maarten J. G. Leening, Frank J. Wolters, Meike W. Vernooij, Blossom C. M. Stephan, M. Kamran Ikram, M. Arfan Ikram

**Affiliations:** 1 Department of Epidemiology, Erasmus MC – University Medical Center Rotterdam, P.O. Box 2040, 3000 CA Rotterdam, The Netherlands; 2 Department of Neurology, Erasmus MC – University Medical Center Rotterdam, Rotterdam, The Netherlands; 3 Department of Radiology and Nuclear Medicine, Erasmus MC – University Medical Center Rotterdam, Rotterdam, The Netherlands; 4 Department of Cardiology, Erasmus MC – University Medical Center Rotterdam, Rotterdam, The Netherlands; 5 Department of Epidemiology, Harvard T.H. Chan School of Public Health, Boston, MA USA; 6 Newcastle University, Newcastle, UK

**Keywords:** Dementia, Alzheimer’s disease, Prediction, Validation

## Abstract

To systematically review the literature for dementia prediction models for use in the general population and externally validate their performance in a head-to-head comparison. We selected four prediction models for validation: CAIDE, BDSI, ANU-ADRI and DRS. From the Rotterdam Study, 6667 non-demented individuals aged 55 years and older were assessed between 1997 and 2001. Subsequently, participants were followed for dementia until 1 January, 2015. For each individual, we computed the risk of dementia using the reported scores from each prediction model. We used the C-statistic and calibration plots to assess the performance of each model to predict 10-year risk of all-cause dementia. For comparisons, we also evaluated discriminative accuracy using only the age component of these risk scores for each model separately. During 75,581 person-years of follow-up, 867 participants developed dementia. C-statistics for 10-year dementia risk prediction were 0.55 (95% CI 0.53–0.58) for CAIDE, 0.78 (0.76–0.81) for BDSI, 0.75 (0.74–0.77) for ANU-ADRI, and 0.81 (0.78–0.83) for DRS. Calibration plots showed that predicted risks were too extreme with underestimation at low risk and overestimation at high risk. Importantly, in all models age alone already showed nearly identical discriminative accuracy as the full model (C-statistics: 0.55 (0.53–0.58) for CAIDE, 0.81 (0.78–0.83) for BDSI, 0.77 (0.75–0.79) for ANU-ADRI, and 0.81 (0.78–0.83) for DRS). In this study, we found high variability in discriminative ability for predicting dementia in an elderly, community-dwelling population. All models showed similar discriminative ability when compared to prediction based on age alone. These findings highlight the urgent need for updated or new models to predict dementia risk in the general population.

**Electronic supplementary material:**

The online version of this article (10.1007/s10654-018-0403-y) contains supplementary material, which is available to authorized users.

## Introduction

Dementia poses an ever-increasing burden on societies worldwide, indicating the urgent need to develop effective therapeutic solutions [1]. Over the last two decades, many pharmacological trials have been conducted to halt or reverse the underlying neurodegenerative process, but have failed to develop disease-modifying therapeutics [2]. Besides the development of treatment strategies in advanced disease stages, there is increasing focus on developing preventive intervention approaches in early disease or asymptomatic states to delay or even prevent the onset of dementia [3, 4]. This shift has been further fueled by recent findings that up to a third of all dementia cases could be prevented if currently known modifiable risk factors were eliminated at a population level [5, 6]. However, a few randomized controlled trials that assessed the efficacy of multi-domain interventions in asymptomatic individuals to prevent cognitive decline or dementia have been inconsistent [7–10]. Moreover, targeting asymptomatic individuals in an unselected population requires expensive trials with large sample sizes and long follow-up duration. In order to make future trials more successful, preventive measures may therefore need to target individuals at high-risk of developing dementia. This requires a valid and reliable method for the identification of high-risk individuals.

Prediction models can be used to discriminate between high- and low-risk individuals, which in turn could result in more tailored selection of individuals for future clinical trials and preventive interventions [11–13]. For dementia, numerous prediction models have been developed over the past years, but for many external validation is lacking [3, 13–15]. A recent systematic review highlighted four models that were most promising for transportability outside the data they were developed on [14]. In order to facilitate practical implementation of any of these prediction models, a direct head-to-head comparison of these prediction models would provide essential information about how the performances compare with each other [15–17]. Therefore, we externally validated four prediction models for dementia in a community-dwelling population.

## Methods

### Ethics statement

The Rotterdam Study has been approved by the Medical Ethics Committee of the Erasmus MC and by the Ministry of Health, Welfare and Sport of the Netherlands, implementing the Wet Bevolkingsonderzoek: ERGO (Population Studies Act: Rotterdam Study). All participants provided written informed consent to participate in the study and to obtain information from their treating physicians.

### Selection of prediction models for external validation

We used a recently published systematic review to identify dementia prediction models [14]. This review identified four models as most promising for practical implementation as preliminary validation was already undertaken on them [18–21]. For this study, we excluded one model as this was developed specifically for individuals with type 2 diabetes [19]. Given that the literature search in this systematic review was last done in March, 2014, we updated the search [14]. The search included articles published between March 17, 2014 until March 31, 2017 in electronic databases (PubMed, Embase, Scopus and, Web of Science). We included articles examining the risk of dementia in non-demented individuals in the general population and constructing a prediction model in which validation was attempted. Combinations of the following terms were used: “dementia”, “risk”, “score”, “assessment”, “prediction”, “model”, and “validation”. Additionally, we searched the reference lists of relevant publications to complement the electronic search strategy. One additional prediction model was identified according to the same criteria used in the systematic review and therefore included in our analyses [22]. Therefore, in total, we included four prediction models in a head-to-head comparison.

### Prediction models included for analysis

The specific models and the studies in which these were developed are briefly described below, with additional data on the original study and model characteristics described in Appendix A. Definitions and distributions of included variables from the corresponding studies are presented in Appendix B, Supplementary Tables 1 and 2.

### Cardiovascular Risk Factors, Aging, and Dementia (CAIDE)

The CAIDE risk score was originally developed in a midlife population (N = 1409) to predict dementia risk during 20 years of follow-up [18]. The model included age (< 47 years: 0 points, 47–53 years: 3 points and > 53 years: 4 points), sex (men: 1 point), education (≥ 10 years: 0 points, 7–9 years: 2 points, 0–6 years: 3 points), hypertension (> 140 mmHg: 2 points), body mass index (> 30 kg/m^2^: 2 points), cholesterol (> 6.5 mmol/L: 2 points), and physical activity (inactivity: 1 point).

### Brief Dementia Screening Indicator (BDSI)

The BDSI was developed and validated using four population-based cohort studies (N = 20,219) to identify individuals aged 65–79 years at increased risk of dementia who could be targeted for cognitive screening in a primary care setting during 6 years of follow-up [20]. The model included age (1 point/year), education (< 12 years: 9 points), body mass index (< 18.5 kg/m^2^: 8 points), presence of diabetes (3 points), history of stroke (6 points), assistance needed with finances or medications (10 points), and depressive symptoms (6 points).

### Australian National University Alzheimer’s Disease Risk Index (ANU-ADRI)

The ANU-ADRI was developed to assess an individual’s risk for late-life Alzheimer’s disease based on self-reported risk factors [23]. The model included 15 risk factors: age and sex (scores stratified on sex, ranging from 0 points for men aged < 65 years to 41 points for women aged ≥ 90 years), educational level (8–11 years: 3 points, > 11 years: 6 points), presence of diabetes (3 points), presence of traumatic brain injury (4 points), presence of depressive symptoms (2 points), high cholesterol (3 points), presence of cognitively stimulating activities (low 0, moderate: − 6 and high: − 7 points), strength of social network (high: 0, medium–high: 1 point, medium–low: 4 points and low: 6 points), smoking (former: 1 point, current: 4 points), alcohol consumption (abstainers: 0 points, and light to moderate − 3 points), level of physical activity (low: 0 points, medium: − 2 points, high: − 3 points), body mass index (normal: 0 points, overweight: 2 points, and obese 5 points), fish intake (< 0.25 serves/week: 0 points, 0.26–2.0: − 3 points, 2.1–4.0: − 4 points, ≥ 4.1: − 5 points), and pesticide exposure (2 points). The model was tested and validated in three population-based cohort studies (N = 5840) [21].

### Dementia Risk Score (DRS)

This model was not identified in the original systematic review, but included for analysis based on our updated literature search. Using The Health Improvement Network (THIN), a database that derived data from routine clinical practice, the DRS was developed and validated to predict a 5-year risk of dementia [22]. The study population was dichotomized based on baseline age for analysis (60–79 years (N = 800,013) and 80–95 years (N = 130,382)). Included predictors per age group are shown in Supplementary Table 1. The risk (P) for an individual aged 60–79 years can be calculated using the following formula: 0.20921 × (age − 65.608) + − 0.00339 × (age − 65.608) × (age − 65.608) + − 0.0616 × (body mass index − 27.501) + 0.002508 × (body mass index − 27.501) × (body mass index − 27.501) + 0.12854 × (female) + 0.13199 × (hypertension) + 0.04477 × (current calender year − 2003.719) + 0.013371 × (depreviation quintile 2) + 0.117904 × (depreviation quintile 3) + 0.201776 × (depreviation quintile 4) + 0.225529 × (depreviation quintile 5) + − 0.06792 × (former smoker) + − 0.08657 × (current smoker) + 0.443535 × (heavy drinking) + 0.833612 × (current depression and/or use of antidepressants) + 0.252833 × (current aspirin use) + 0.577207 × (history of stroke or TIA) + 0.220728 × (history of atrial fibrillation) + 0.286701 × (history of diabetes). With a baseline hazard of 0.9969. The predicted 5-year risk as a percentage is then calculated as follows: 100 × [1 − S^exp(P)^].

### Study population of the external validation cohort

Participants were recruited within the Rotterdam Study, a prospective population-based cohort study. In 1990, all residents aged 55 and older residing in Ommoord, a district of Rotterdam, the Netherlands, were invited. Of the 10,215 invited inhabitants, 7983 (78%) agreed to participate in the baseline examination. In 2000, the cohort was extended: all residents aged 55 and older of the same district were invited, except for the participants that were already in the original cohort. Of the 4472 invitees, 3011 (67%) agreed to participate. Follow-up examinations take place every 3–4 years [5, 24].

Analyses of this study are based on data obtained from the third follow-up round of the original wave undertaken 1997–1999 (N = 4797) and the first round of the extended wave undertaken 2000–2001 (N = 3011). While these study waves had different initiation dates, they were similar in design and participants came from the same source population, i.e. Ommoord, a suburb of Rotterdam, the Netherlands. After excluding participants who did not complete the interview and research center visit in these rounds (N = 873), had dementia or insufficient screening for dementia at baseline (N = 99), did not provide informed consent to access medical records and hospital discharge letters (N = 149), or if there was no follow-up due to logistic reasons (N = 20), 6667 participants were included for analysis in this study, of whom 3983 participants came from the original wave (83.0% of surviving participants), and 2684 participants (89.1%) from the extended wave. Results have been reported to conform with the TRIPOD statement [25].

### Assessment of dementia

Participants were screened for dementia at baseline and subsequent center visits with the Mini-Mental State Examination and the Geriatric Mental Schedule organic level [5]. Those with a Mini-Mental State Examination score < 26 or Geriatric Mental Schedule score > 0 underwent further investigation and informant interview, including the Cambridge Examination for Mental Disorders of the Elderly. All participants also underwent routine cognitive assessment. In addition, the entire cohort was continuously under surveillance for dementia through electronic linkage of the study database with medical records from general practitioners and the regional institute for outpatient mental health care. The information from in-person screening was supplemented by data from the electronic linkage of the study database with medical records from all general practitioners and the regional institute for outpatient mental health care. In the Dutch healthcare system, the entire population is entitled to primary care that is covered by their (obligatory) health insurance. The general practitioner functions as a ‘gate-keeper’ for referral to secondary and tertiary care providers, who are required by law to report back to the referring general practitioner about test results and clinical diagnoses. With this linkage, the entire cohort is thus continuously monitored for detection of interval cases of dementia or cognitive disturbances between center visits. Study physicians biannually evaluate all records, and combine information from medical records with in-person screening to draw up individual case reports. In these reports, the physicians covered all gathered relevant information to establish the presence, probability and subtype of dementia. Available information on cognitive testing and clinical neuroimaging was only used if required for diagnosis of dementia subtype. Available information on cognitive testing and clinical neuroimaging was used when required for diagnosis of dementia subtype. A consensus panel led by a consultant neurologist established the final diagnosis according to standard criteria for dementia (DSM-III-R) and Alzheimer’s disease (NINCDS–ADRDA). Follow-up for incident dementia was near-complete (97.5% of potential person-years) until 1 January, 2015 [26]. Within this period, participants were censored at date of dementia diagnosis, death, loss to follow-up, or 1st January 2015, whichever came first.

### Assessment of predictors

The predictors used in this validation study are based on the component variables included in the different published risk prediction models and are described in detail in Supplementary Appendix B. We had to make a few adjustments to the included variables due to different measurement methods as compared to the original models. For the CAIDE model, we measured physical activity using the Zutphen physical activity questionnaire [27]. We did not have data available on the frequency of physical activities per week, therefore we defined being physically active based on a minimum of ≥ 40 min of exercise per week with a metabolic equivalent of task (MET) intensity of ≥ 4. For the ANU-ADRI model, we reduced the social engagement predictor from five to three domains in our model (based on marital status, living status, and loneliness). We were unable to include pesticide exposure and cognitive activity in this model, as these are not systematically measured within the Rotterdam Study. In the DRS model, the Townsend deprivation index was used to indicate neighborhood deprivation. This index is uniquely used in the United Kingdom. We therefore constructed a similar composite score based on living status, marital status, and loneliness to emulate this index in our sample. Use of such composite scores of socio-demographic domains to summarize neighborhood deprivation have been used and validated previously [28]. In addition, the DRS included anxiety disorders, but we did not have questionnaires available to assess anxiety symptoms. Therefore, we defined anxiety symptoms as present if participants used anxiolytic drugs (Anatomical Therapeutic Chemical Classification codes N05B).

### Statistical analysis

We evaluated two measures of model performance: discrimination and calibration. Discrimination refers to the capability of a risk score to correctly differentiate between two participants, one who will develop the outcome during follow-up and one who will not [29]. We used C-statistics to assess the discriminative ability of the models. Calibration is the agreement between the risks predicted by the model and the observed frequencies of the outcomes under study, which we evaluated using calibration plots [29].

We present three sequential analyses to compare the discriminative performance of the models in the validation cohort. First, we evaluated the performance of the models in the age ranges for which they were originally designed. We computed the risk scores for each participant exactly as published in the original publication for each prediction model. If this was not provided, we used the linear predictor, which represents the sum of all regression coefficients.

Second, we validated all models in the entire study population to directly compare the performance of all models across the entire age range. We needed to perform a few adjustments to the original risk scores to be able to make this direct comparison, because some of the included prediction models were designed for specific age ranges, which were narrower than our study population. The BDSI was originally developed for a population aged 65–79 years old, ranking individuals with 1 point extra per year increase in age. We therefore extrapolated the corresponding BDSI score for participants outside this range in this validation study using the same point increase in age. The DRS used two separate risk equations for different age strata. As these equations did not capture the entire age range within this validation study, we used the risk equation designed for a 60–79-year-old population for all participants below 79 years of age and the risk equation for a 80–95-year-old population for participants above 80 years of age for this analysis. Third, to quantify the added predictive value of other predictors in the models, we evaluated the predictive accuracy of the models based on age alone, the strongest risk factor for dementia, and based on all risk factors with the exception of age.

Predicted time horizons differed across the original studies, ranging from 5 to 20 years. For all analyses, we focused on 10-year dementia risk in all four models to facilitate a fair comparison. We additionally studied the performance of the models with follow-up of 2, 5, and 15 years. We truncated the follow-up for participants with longer follow-up time than these horizons.

To assess calibration, we constructed 10-year risk calibration plots and evaluated the intercept and calibration slope of these plots to test the goodness-of-fit of the models [29]. Furthermore, we recalibrated original logistic regression models by updating the intercept. For Cox models, we updated the baseline survival function and used the mean predictor values of the validation study to account for possible differences in disease incidence and risk factor distribution [30, 31].

### Sensitivity analyses

In sensitivity analyses, (1) we assessed the predictive accuracy for Alzheimer’s disease specifically, (2) we repeated the analyses of a 10- and 15-year time horizon with exclusion of participants with less than 4 years of dementia-free follow-up to reduce the possibility of reverse causality (i.e. prodromal dementia leading to a higher risk score), and (3) we stratified on age (80 years) at baseline, given the steep increase in incidence of dementia beyond this age in order to further investigate the performance of the models in different age strata.

Missing data on covariates were imputed using 5-fold multiple imputation, based on all predictors, outcome status, and follow-up time. All analyses were done using IBM SPSS (version 21.0) and R, CRAN version 3.3.2 (rms [32], val.prob.ci.2 packages [33]).

## Results

In Table 1 the baseline characteristics of the study sample are presented. Missing data on the included predictors for this validation study was relatively low (< 8.7% missing), except for head trauma (40.9%) and fish servings per week (48.1%) as these were only measured in one of the two included study waves. The mean age was 69.1 years (standard deviation 8.2) and 57.0% were women. 2466 (37%) of all participants were middle-aged (< 65 years old), of whom 1377 (55.8%) were women. The median follow-up time for the full sample was 13.2 years (interquartile range 10.1–16.3), with a median follow-up of 12.5 years (8.4–16.6) for the original wave and 13.6 years (11.9–15.3) for the extended wave, respectively. During a total follow-up of 75,581 person-years, 867 participants developed dementia, 696 of whom developed Alzheimer’s disease. This corresponds to a crude incidence rate for all-cause dementia of 11.5 per 1000 person-years.

**Table 1:** Baseline characteristics of the validation cohort

	All participants N = 6667	Missing data (%)	Original study wave N = 3983	Missing data (%)	Extended study wave N = 2684	Missing data (%)
Age, years	69.1 (8.2)	0	72.2 (7.0)	0	64.6 (7.9)	0
Women	3787 (56.8%)	0	2306 (57.9%)	0	1481 (55.2%)	0
Body mass index, kg/m^2^	27.0 (4.0)	1.5	26.9 (4.0)	1.3	27.3 (4.0)	1.6
Systolic blood pressure, mmHg	143 (21)	0.9	144 (21)	0.4	143 (22)	1.4
Education, years	11.4 (3.6)	1.8	11.0 (3.6)	1.4	12.2 (3.5)	2.3
Alcohol use	5477 (82.2%)	1.0	3247 (81.5%)	0.7	2230 (83.1%)	1.4
Smoking		0.9		0.7		1.3
Never	2059 (30.9%)		1289 (32.4%)		770 (28.7%)	
Former	3222 (48.3%)		1952 (49.0%)		1270 (47.3%)	
Current	1323 (19.8%)		715 (18.0%)		608 (22.7%)	
Total cholesterol, mmol/L	5.80 (0.98)	4.1	5.8 (0.98)	3.9	5.8 (0.98)	4.5
High-density lipoprotein cholesterol, mmol/L	1.39 (0.39)	5.2	1.39 (0.40)	5.7	1.37 (0.37)	4.5
Total-to-HDL-cholesterol ratio	4.47 (1.32)	5.2	4.47 (1.33)	5.7	4.47 (1.30)	4.5
Leisure time physical activity, MET-hours (IQR)*	77.0 (48.7–105.2)	4.2	78.2 (48.2–108.2)	0.1	75.1 (48.4–101.8)	8.2
History of type 2 diabetes	717 (10.8%)	7.3	437 (11.0%)	7.3	280 (10.4%)	7.3
History of stroke	244 (3.7%)	1.0	156 (3.9%)	1.0	88 (3.3%)	1.0
History of TIA	194 (2.9%)	3.0	112 (2.8%)	3.0	82 (3.1%)	3.2
History of head trauma with unconsciousness	442 (6.6%)	40.9	442 (11.1%)	1.1	–	–
History of atrial fibrillation	335 (5.0%)	2.8	256 (6.4%)	2.4	79 (2.9%)	3.0
Depressive symptoms	508 (7.6%)	4.2	241 (6.1%)	5.7	267 (9.9%)	2.1
Social engagement†		0.4		0.6		0.2
High	19 (0.3%)		0 (0%)		19 (0.7%)	
Medium–high	661 (9.9%)		174 (4.4%)		487 (18.1%)	
Medium–low	4827 (72.4%)		2928 (73.5%)		1899 (70.8%)	
Low	1130 (16.9%)		855 (21.5%)		275 (10.2%)	
Fish servings per week		48.1		12.9		–
> 4.1	15 (0.2%)		15 (0.2%)		–	
2.1–4.1	88 (1.3%)		88 (1.3%)		–	
0.26–2.0	1837 (27.6%)		1837 (27.6%)		–	
≤ 0.25	1529 (22.9%)		1529 (22.9%)		–	
Needs help with finances or medications	1180 (17.7%)	7.1	935 (23.5%)	6.8	245 (9.1%)	8.8
Use of antihypertensive drugs	1551 (23.3%)	4.8	962 (24.2%)	4.8	589 (21.9%)	4.6
Use of anxiolytics	790 (11.8%)	0	543 (13.6%)	0	247 (9.2%)	0
Use of aspirin	1158 (17.4%)	0	780 (19.6%)	0	378 (14.1%)	0
Use of NSAIDs (excluding aspirin)	569 (8.5%)	0	331 (8.3%)	0	238 (8.9%)	0

*N* number of people at risk, *lipid ratio* ratio between total and high-density lipoprotein cholesterol, *MET* metabolic equivalent of task, *IQR* interquartile range, *TIA* transient ischemic attack, *NSAIDs* non-steroidal anti-inflammatory drugs

Data are shown for non-imputed data. Values are counts (valid percentages) or means (standard deviation)

*Presented as median (interquartile range), because of skewed distribution

†Used as a social deprivation index for the dementia risk score

### Discrimination

As the BDSI and DRS models were designed for a specific age range, we first evaluated their performance in this age range using a comparable predicted time horizon. The models showed slightly attenuated discriminative ability in this validation study (C-statistic: 0.69 (95% CI 0.64–0.73) for the BDSI and 0.77 (0.72–0.81) for the DRS) compared to the original development samples (C-statistics: ranging from 0.68 to 0.78 for BDSI and 0.84 (0.81–0.87) for the DRS).

Table 2 shows the discriminative ability for all models across the entire age range within our validation sample. The BDSI, ANU-ADRI, and DRS showed the highest C-statistics at 10-year horizons. These models all contained the predictors age, history of diabetes, and presence of depressive symptoms. Using different predicted horizons, C-statistics ranged from 0.55 for the CAIDE to 0.84 for the DRS model, both at predicting a 2-year risk of dementia.

**Table 2:** Discriminative ability for all-cause dementia

Prediction model	C-statistics at various follow-up horizons (95% CI)			
	2 years n/N = 63/6667	5 years n/N = 233/6667	10 years n/N = 515/6667	15 years n/N = 847/6667
CAIDE	0.49 (0.42–0.56)	0.54 (0.50–0.58)	0.55 (0.53–0.58)	0.55 (0.53–0.57)
Age only	NA	NA	NA	NA
Without age	0.49 (0.42–0.56)	0.54 (0.50–0.58)	0.55 (0.53–0.58)	0.55 (0.53–0.57)
BDSI	0.83 (0.75–0.90)	0.80 (0.76–0.84)	0.78 (0.76–0.81)	0.76 (0.74–0.78)
Age only	0.83 (0.76–0.90)	0.81 (0.78–0.85)	0.81 (0.78–0.83)	0.79 (0.77–0.81)
Without age	0.64 (0.57–0.71)	0.63 (0.59–0.66)	0.60 (0.58–0.63)	0.59 (0.57–0.61)
ANU-ADRI	0.81 (0.77–0.86)	0.78 (0.76–0.81)	0.75 (0.74–0.77)	0.70 (0.69–0.72)
Age only	0.83 (0.79–0.87)	0.80 (0.77–0.82)	0.77 (0.75–0.79)	0.72 (0.71–0.74)
Without age	0.56 (0.49–0.64)	0.51 (0.47–0.55)	0.52 (0.49–0.54)	0.51 (0.49–0.53)
DRS	0.84 (0.77–0.92)	0.82 (0.78–0.86)	0.81 (0.78–0.83)	0.79 (0.77–0.81)
Age only	0.83 (0.76–0.90)	0.81 (0.78–0.85)	0.81 (0.78–0.83)	0.79 (0.77–0.81)
Without age	0.63 (0.56–0.70)	0.58 (0.54–0.62)	0.57 (0.55–0.60)	0.55 (0.53–0.57)

*CI* confidence interval, *n* number of cases, *N* number of people at risk, *CAIDE* cardiovascular risk factors, aging, and dementia study, *NA* not applicable, *BDSI* brief dementia screening indicator, *ANU-ADRI* Australian National University Alzheimer’s Disease Risk Index and, *DRS* dementia risk score

Importantly, calculating the C-statistics based on the age component of the models only, showed nearly identical discriminative abilities compared with the full models for all predicted horizons. This applied for the specific age ranges (Supplementary Table 3) and for the entire age range within our sample (Table 2). Conversely, excluding the age component from the risk scores showed very poor predictive abilities for all models (Table 2). Discriminative ability based on the age component alone could not be calculated for the CAIDE model as all participants within this validation study were in the oldest age group according to this model. Hence, the C-statistics for this model can be best compared to the C-statistics of the other models excluding age. With the exception of CAIDE, discriminative ability was inversely related to the predicted horizon, with the highest C-statistics when calculating short-term dementia risks.

### Calibration

**Fig. 1:**
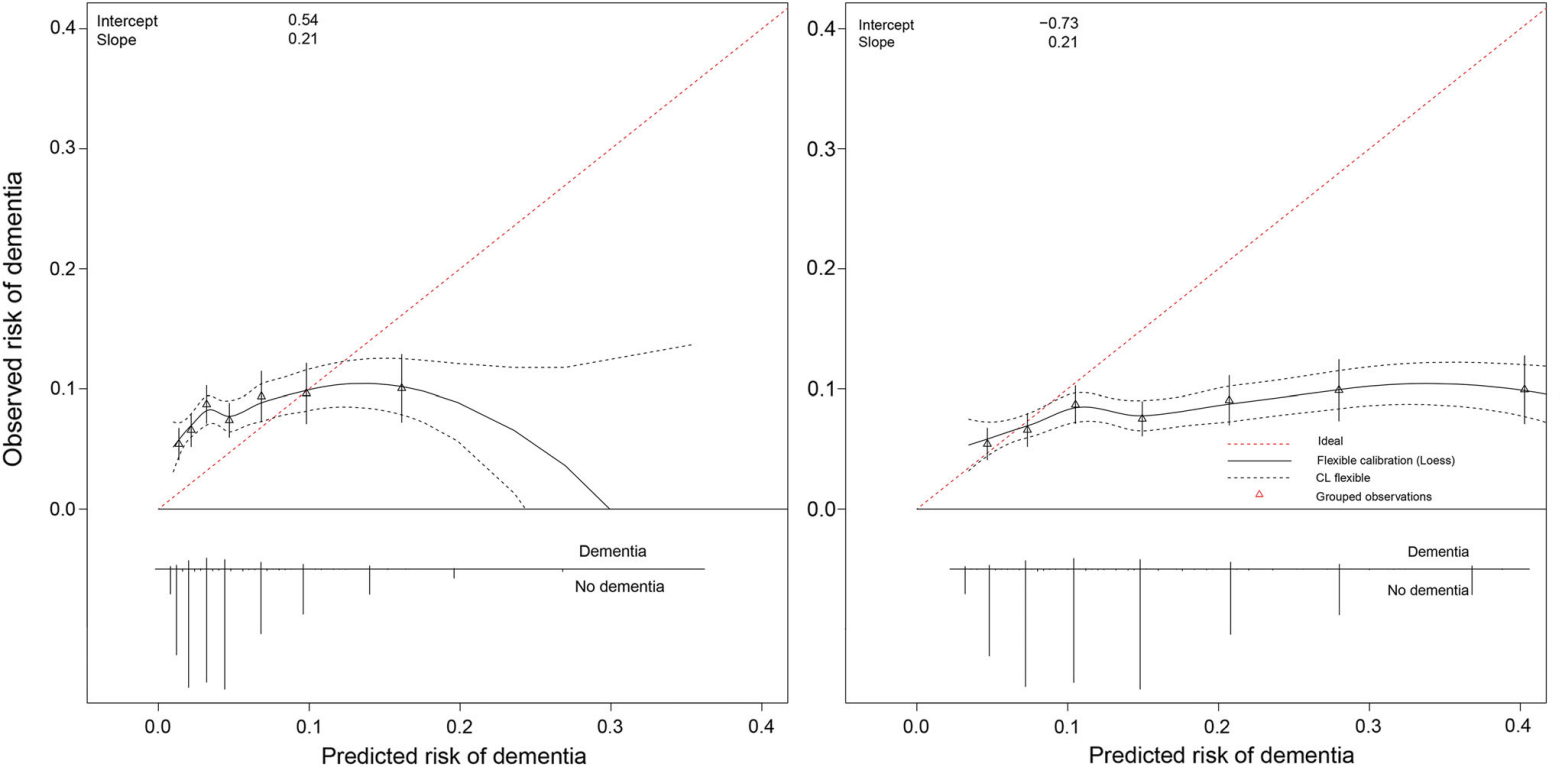
Calibration plots of the original (left) and recalibrated (right) Cardiovascular Risk Factors, Aging, and Dementia (CAIDE) model to predict risk of dementia. In case of perfect calibration all groups of predicted probabilities fit close to the red diagonal line, corresponding to an intercept of 0 and a slope of 1 for the calibration plot. Vertical lines in grouped observations represent 95% confidence intervals. (Color figure online)

**Fig. 2:**
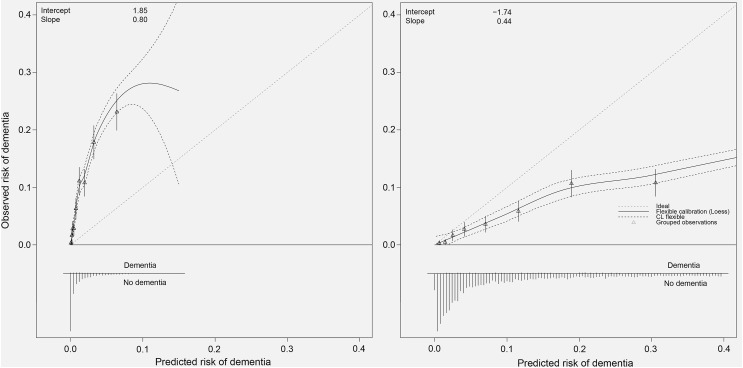
Calibration plots of the original (left) and recalibrated (right) Dementia Risk Score (DRS) model to predict risk of dementia. In case of perfect calibration all groups of predicted probabilities fit close to the red diagonal line, corresponding to an intercept of 0 and a slope of 1 for the calibration plot. Vertical lines in grouped observations represent 95% confidence intervals. (Color figure online)

The required absolute risk equations needed to construct calibration plots were only reported for the CAIDE and DRS models (Supplementary Appendix C). In Fig. 1 the calibration plots are shown for the original and recalibrated CAIDE models. The CAIDE model tended to systematically underestimate the risk of dementia (‘calibration-in-the-large’). The recalibrated CAIDE with updated intercept still showed poor calibration (intercept = − 0.73, calibration slope = 0.21), reflecting the poor discriminative ability of the model in the validation sample. The DRS model also tended to underestimate risks (Fig. 2). The recalibrated DRS model using an updated dementia incidence rate and mean values based on the Rotterdam Study population, performed better, but still indicated that predictions were too extreme, particularly for those at high predicted risk (Fig. 2).

### Sensitivity analyses

All models showed similar discriminative performance based on the age component of the risk score alone. This applied for all sensitivity analyses: for predicting Alzheimer’s disease specifically (Supplementary Table 3), in participants with four or more years of dementia-free follow-up (Supplementary Table 4), and in participants below and above the age of 80 at baseline (Supplementary Table 5).

## Discussion

In this external validation study, we identified four dementia prediction models based on a previously published systematic review complemented by an updated literature search and showed that these models have widely varying accuracy for predicting dementia in an elderly, community-dwelling population. Importantly, in all models age was the driving factor for the discriminative ability. Other risk factors included had marginal contributions above and beyond age. Our results indicate that established risk factors for dementia that are currently included in these models have limited added value in dementia prediction above and beyond age in the general population. Our results were consistent in several analyses, even when we compared the performance of the models based on the age component alone with the performance of the full models in the specific age ranges on which those models were originally derived from.

To our knowledge, there is currently no other head-to-head comparison of multiple dementia prediction models in the same study population. Other studies only validated one prediction model or compared the novel model with one other prediction model in the same study population [21, 34].

We will briefly consider several differences between the original studies and this validation study, which may have influenced the observed performance in this study. The CAIDE dementia risk score was originally developed for a midlife population and showed poor transportability to our elderly population. The poor performance in this study is in line with a previous study which assessed the performance of the CAIDE risk score in three elderly population-based cohorts [21]. This reflects the importance of age. Indeed, in the Rotterdam Study, all participants received the highest score for age and it was therefore not possible to discriminate participants based on the age component of the risk score alone. In addition, this poor performance may also be due to specific midlife risk factors for dementia, such as high body mass index and cholesterol level which are found to have inverse (i.e. protective rather than increasing risk) associations with dementia in older age groups [35, 36]. Given these considerable differences, we note that the application of the CAIDE model in older adults is limited. The results of the CAIDE model should therefore be interpreted with caution, yet we also note that the CAIDE model is increasingly being used in older populations to select high-risk individuals for clinical trials [7] and to conduct stratified analyses in high-risk individuals using this score [9]. Results from this study provide important insights in these transportability issues and quantify the predictive ability of this model in these populations.

The BDSI model was originally developed for a 65–79 year old population to identify individuals who could be targeted for cognitive screening during 6-years of follow-up. We extrapolated the age component of the original model to be able to compare this model with other models across the entire age range. This may have overestimated the importance of age in this adjusted model. Nevertheless, the full model showed similar discriminative performance compared with the model based on the age component alone when we evaluated its performance in the age range it was originally designed for with use of a comparable predicted time horizon [20].

The ANU-ADRI model was originally developed to assess an individual’s risk for late-life Alzheimer’s disease. Although we compared this model with other models in our main analysis to predict the risk of all-cause dementia, similar results were seen for Alzheimer’s disease.

The DRS was developed and validated using data derived from routine clinical practice. For the original model, two separate risk equations were developed based on an individual’s age at baseline (60–79 vs. 80–95 years). In this validation study, we evaluated the performance of this model using these two risk equations for our entire study population, which was broader than the age range for which they were originally developed. This may have affected our results, but as with the BDSI, the model based on the age component alone showed comparable predictive accuracies with the full model when we restricted our analyses to the specific age ranges for which the risk equations were designed.

All models included established risk factors for dementia and most often assigned the highest weight to age, reflecting age as most important risk factor for the occurrence of dementia. Beyond age, however, other risk factors appear not to be as important when used as risk predictors for dementia in an elderly population. Most risk factors are not very specific for the occurrence of dementia, whereas risk factors that are also useful as risk predictors need to be very strongly associated with the disease to provide additional predictive value. Indeed, we find that various factors that have been deemed risk factors for dementia do not add to the prediction of dementia beyond age alone. Hence, our current analyses are not in conflict with these factors being risk factors for dementia, but additionally show that these risk factors do not provide additional predictive utility beyond age alone. On the other hand, age reflects a cumulative risk index of exposure to various risk factors over time. Chronological age therefore probably yields a summary of predictive information derived from these factors that accumulate over the lifespan, thus covering most of their predictive value. Moreover, when an established risk factor is considered as a risk predictor, there is need for sufficient variation within the population in such risk factor in order to successfully discriminate between high and low risk groups [37]. For instance, pesticide exposure is associated with incident dementia [38], but in the general population only few individuals have been exposed to these substances. Therefore, at population level the inclusion of such a predictor has a very limited yield.

Our results furthermore indicate that the predictive accuracy of all models is poor in participants aged 80 years or older. This may be explained by the fact that although the absolute incidence of dementia increases steeply with increasing age, the relative increase in dementia incidence is higher in younger (aged 55–79 years) than in older participants (aged 80 years or older) [20, 39]. A higher predictive value of an increase in age in the younger compared to the older group of participants was therefore not unexpected. Conversely, the incorporated midlife cardiovascular risk factors in these models will contribute more to dementia risk for younger participants at elderly ages. These results emphasize the need for more advanced modelling of age-specific effects (e.g. non-linear or interaction terms) and warrant further development of age-stratified models. This approach is probably a more likely key to success in dementia risk prediction modelling, as age is the main driver of dementia risk. In addition to more adequate modelling of the effects of age and its interactions with risk factors, future models could take other useful risk predictors for dementia into account. These are probably early minor symptoms of disease, such as subjective memory complaints. For more augmented models, markers of subclinical neurodegeneration could be considered, such as hippocampal atrophy, to improve model performance in a more specific, clinical setting [40].

Altogether, this study shows that using age alone has similar predictive accuracy for the occurrence of dementia compared to risk models incorporating demographic, health, and lifestyle risk factors. These findings highlight the limited added value of other predictors currently included in these models in dementia prediction above and beyond age in an elderly population. Additionally, we mention several methodological considerations that deserve further attention when developing dementia prediction models. First, it is informative to assess and report the performance of the full model compared to a model based on age alone prior to and in addition to external validation. Second, given distinct differences in risk factor distributions between men and women, it may be of additional value to explore the additional value of sex-specific models. Third, while model discrimination was appropriately addressed in most of these models, equations to calculate absolute risks were often not provided—limiting required limiting opportunities for proper validation, and eventually hampering clinical translation. Finally, dementia models included in this validation study did not account for the competing risk of death from other causes, subsequently inflating apparent dementia risk predictions. The risk of these competing events is substantial, given the late-life onset of dementia in the general population and future models should therefore take this into account [41]. Strengths of this study include the large sample size and number of events, detailed assessment of dementia and the wide range of systematically collected covariates, making this comparison possible. Several general methodological considerations need to be taken into account for proper interpretation of our findings. First, although we tried to compute the risk scores of the models exactly as they were reported, we had to make several adjustments. These include some minor adjustments that were made to variable definitions. Nevertheless, these deviations most likely represent a good approximation of performance based on other data that, similarly, will likely not map directly onto the original variables. Second, there was no data or surrogate marker available in this study on pesticide exposure and cognitive activity, two predictive variables in the ANU-ADRI risk score. This may have underestimated the performance of this score in the Rotterdam Study. Third, selective attrition cannot be ruled out, yet we believe that given the high response figures of each study wave (83.0 and 89.1%) along with a virtually-complete follow-up (97.5%), it is very unlikely that this may have influenced our results. Finally, as our study population consists of elderly participants of predominantly Caucasian descent (97.7%), we cannot generalize our findings individuals up to 55 years of age or to other ethnicities.

## Conclusions

In conclusion, this validation study shows that the performance of four models for predicting dementia in an elderly community-dwelling population using age alone was nearly identical compared to the full prediction models. Discriminative abilities of the models varied largely and was very age-dependent. Transportability of the predicted risks was generally poor. These findings highlight the importance of age in the assessment of dementia risk and indicate a need for improvement and refinement in risk factor measurement and model development to inform prediction above and beyond the risk from age alone.

## Electronic supplementary material

Below is the link to the electronic supplementary material.

Supplementary material 1 (DOCX 113 kb)
