# Supplementary material for: External validation of four dementia prediction models for use in the general community-dwelling population: a comparative analysis from the Rotterdam Study
Source: Eur J Epidemiol. 2018 May 8;33(7):645–55. doi: 10.1007/s10654-018-0403-y (PMC6061119; doi:10.1007/s10654-018-0403-y)
Supplement: Supplementary file 1 — Supplementary material 1 (DOCX 113 kb) [file 10654_2018_403_MOESM1_ESM.docx]

**External validation of four dementia prediction models for use in the general community-dwelling population: a comparative analysis from the Rotterdam Study**

**Supplementary material**

**Appendix A.** Original study and model characteristics ……………….……...……...............…..2

## Appendix B. Definitions, distributions and descriptions of predictors…………….................…..5

## Appendix C. Full model equations for the risk of dementia as applied in the validation cohort……………………………………………………………….……....................................13

## Appendix D. Supplementary tables…………………………………………..…..……………..14

**Appendix E.** TRIPOD Checklist: Prediction Model Validation……………….………………..19

## Appendix A.

**Original study and model characteristics**

### Cardiovascular Risk Factors, Aging, and Dementia (CAIDE)

The CAIDE risk score was originally developed in a midlife population (N=1409) to predict dementia risk during 20 years of follow-up (1). Dementia was diagnosed according to criteria of the Diagnostic and Statistical Manual of Mental Disorders (Fourth Edition) (DSM-IV). The model included age, gender, education, hypertension, body mass index, cholesterol, and physical activity. In an additional model, *APOE*-ε4 status was added. Based on the published C-statistics, the model showed a discriminative performance of 0.77 (95% confidence interval (CI), 0.71-0.83). Addition of *APOE*-ε4 carrier status showed nearly identical results 0.78 (95% CI, 0.72-0.84). In a validation study, the original CAIDE risk score replicated well with a C-statistic of 0.75 during almost 40 years of follow-up in a midlife population (N=9408) using a retrospective health survey study (2).

### Brief Dementia Screening Indicator (BDSI)

The BDSI was developed and validated using four population-based cohort studies (N=20 219) to identify individuals aged 65-79 years at increased risk of dementia who could be targeted for cognitive screening in a primary care setting during 6 years of follow-up (3). Minimum requirements for dementia diagnosis were cognitive impairment in at least two domains and sufficient severity to interfere with daily function. The model included age, education, body mass index, presence of diabetes, history of stroke, assistance needed with finances or medications, and depressive symptoms. The discriminative ability measured using the C-statistic ranged from 0.68 (95% CI, 0.65-0.72) to 0.78 (95% CI, 0.72-0.83) across the four cohorts. Calibration was assessed graphically by evaluating the agreement between predicted and actual risks over deciles of predicted risk, which suggested reasonable correspondence, particularly for higher deciles of predicted risk (3).

### Australian National University Alzheimer’s Disease Risk Index (ANU-ADRI)

The ANU-ADRI was developed to assess an individual’s risk for late-life Alzheimer’s disease based on self-reported risk factors (4). The model included 15 risk factors (Supplementary Table 1). The model was tested and validated in three population-based cohort studies (N=5,840) (5). Dementia was diagnosed by experienced physicians using DSM-III criteria. The National Institute of Neurological and Communicative Disorders and Stroke–Alzheimer’s Disease, and Related Disorders Association (NINCDS-ADRDA) criteria were used for Alzheimer’s disease. The C-statistics ranged from 0.64 (95% CI, 0.60-0.68) to 0.74 (95% CI, 0.71-0.77). Although the risk index was originally developed for Alzheimer’s disease, C-statistics showed similar results for all-cause dementia (5). Data on model calibration was not reported.

### Dementia Risk Score (DRS)

The DRS was developed in the United Kingdom using The Health Improvement Network (THIN), a large database that derived data from routine clinical practice (6). The study population was dichotomized based on baseline age for analysis (60-79 years (N=800,013) vs 80-95 years (N=130,382)). ICD-10 codes and symptom descriptions were used to identify recorded diagnoses of dementia. Several predictors were identified using Cox proportional hazards regression models depending on the age group, using backward elimination (final included predictors are shown in Supplementary Table 1). The validation study was conducted using the same database, on a random subsample of other general practices. Eventually, for each age group separate risk equations were reported. Discrimination in the validation sample showed a C-statistic of 0.84 (95% CI, 0.81-0.87) for the group aged 60-79 years. In this group, the model suggested good calibration with a calibration slope of 0.95 (95% CI, 0.93-1.02). In the participants aged 80-95 years at baseline, the model showed poorer performance in terms of discrimination (C-statistic 0.56, 95% CI, 0.55-0.58) and calibration (calibration slope 1.04, 95% CI, 0.89-1.18).

## Appendix B.

## Definitions, distributions and descriptions of predictors

## Table 1. Definitions of predictors per included model and validation sample

| **Variables** | **Validation Study** | | | | |
| --- | --- | --- | --- | --- | --- |
|  | **Cardiovascular Risk Factors, Aging and Dementia (CAIDE) Study(1)** | **Brief Dementia Screening Indicator (BDSI)(3)** | **Australian National University Alzheimer’s Disease Risk Index (ANU-ADRI)(5)** | **Dementia Risk Score (DRS)(6)** | **Rotterdam Study** |
| Age at baseline | Self report | Self report | Self report | Based on general practices data | Based on municipality data |
| Gender | Self report | NA | Self report | Based on general practices data | Based on municipality data |
| Obesity | Body mass index (obesity: >30kg/m^2^ ) | Body mass index (obesity ≥30kg/m^2^ ) | Body mass index (if aged<60 years) | Body mass index | Body mass index |
| Hypertension | Systolic blood pressure >140 mmHg, measured 5 min after participant had been seated from the right arm | NA | NA | Current use of antihypertensive medication. | Systolic and diastolic blood pressure was assessed at the right arm and the mean of two measurements was used in the analyses Antihypertensive use  was continuously gathered, using linkage of pharmacy records |
| Education | Self report. Scale was created using number of years. | Self report, (≥12 years of education as reference) | Scale was created using number of years | NA | Scale was created using number of years. Categorized according to the models |
| Alcohol | NA | NA | Categories were calculated according to NHMRC guidelines using number of drinks per week | History of heavy alcohol use (more than 56 units per week for men and more than 49 units per week for women) or a code entry in their medical records indicating an alcohol problem | Categories were calculated according to NHMRC guidelines using number of drinks per week based on a questionnaire: “How many units of alcohol do you consume during one week?” |
| Smoking | NA | NA | Current smoking, ever and never | Smoking status, divided into current, ex-smoker and non-smoker, up to 5 years prior to baseline. | Current smoking, ever and never. For the CAIDE-extended model current and ever smokers were merged |
| Hypercholesterolemia | Total serum cholesterol (auto analyzer), >250.9 mg/dL (>6.5 mmol/L) | NA | Normal: <6.2 mmol/L, high: > 6.2 mmol/L | NA | The lipids were measured in fasting serum. Hypercholesterolemia was defined according to the definitions used in the different models |
| Physical activity | Being active or inactive on the basis of the frequency of leisure time physical activity. Engaging minimal 20-30 minutes at least twice a week and causing sweating and breathlessness were regarded as active, others as inactive | NA | International Physical Activity Questionnaire (IPAQ) categories were calculated using MET-value. | NA | Zutphen physical activity questionnaire. Sum of leisure time activity in hours and Metabolic equivalent of task (MET-value). Categories were calculated based on IPAQ using MET-values.  For the CAIDE model, we specified participants as physically active if they exercised ≥ 40 minutes/week with a MET intensity of ≥4 |
| History of type 2 diabetes | NA | Medical history of type 2 diabetes | History of diabetes and medication | Based on medical diagnoses, extracted from general practices data at any time prior to baseline | Type 2 diabetes was defined as fasting serum glucose levels ≥7.0 mmol/L, non-fasting serum glucose levels ≥11.0 mmol/L (if fasting samples were not available), or the use of blood glucose-lowering therapy |
| History of stroke | NA | Medical history | NA | Based on medical diagnoses, extracted from general practices data. Either a stroke or a TIA in medical history at any time prior to baseline | At baseline, history of stroke or TIA was assessed by interview and verified using medical records. Study participants were continuously followed up for occurrence of incident stroke, based on information collected continuously from medical records of general practitioners and neurologists. |
| History of traumatic brain injury | NA | NA | History for TBI with loss of consciousness | NA | Questionnaire. “Did you ever have a serious head trauma or a concussion?” and “Were you ever unconscious because of a head trauma?” |
| History of atrial fibrillation | NA | NA | NA | Atrial fibrillation at any time prior to baseline | Atrial fibrillation at any time prior to baseline. Prevalent AF was diagnosed with ECG and participants were continuously monitored with linkage of medical records |
| Depressive symptoms | NA | Current usage of antidepressants or reporting that “everything was an effort” for 3 days or more a week for the past week using the CES-D | CES-D (20 item) > 16 was used as cut-off | Current (in 12 months prior to baseline) depression diagnosis or treatment with antidepressant medication. | CES-D questionnaire and use of antidepressants (for the BDSI and DRS model).  Depression was defined as CES-D (20 item) > 16. |
| Social network and engagement | NA | NA | 5 domains (marital status, size of social network, quality of social network, level of social activities and living arrangements) | NA | Social and network engagement was constructed based on three domains, using questionnaires on: 1.Marital status 2. Living arrangements: Do you live alone, with your spouse or with others? 3. Loneliness, using a subset of the CES-D questionnaire: “During the past week I felt lonely” |
| Social deprivation | NA | NA | NA | Using the Townsend index: a combined measure of owner occupation, car ownership, overcrowding, and unemployment based on postal code. The score ranges from 1 (least deprived) to 5 (most deprived) | Social deprivation was emulated based on three domains, using questionnaires on: 1.Marital status 2. Living arrangements: “Do you live alone, with your spouse or with others?” 3. Loneliness, using a subset of the CES-D questionnaire: “During the past week I felt lonely” |
| Fish servings per week | NA | NA | National Cancer Institute FFQ, <25 pp week (reference) [serves per week] | NA | Using a validated FFQ, participants were specifically asked to indicate the frequency, amount, and kind of fish they had eaten. Portions per week were calculated |
| Needs help, money/medications | NA | Self report or ask present family member: “Do you need help from others to manage money or medications?” | NA | NA | Using a questionnaire. “[If you had to take medicine, can you do it?](https://epi-wiki.erasmusmc.nl/wiki/ergowiki/index.php?title=If_you_had_to_take_medicine,_can_you_do_it%3F&action=edit&redlink=1) and “Can you manage your own financial matters?” |
| Anxiety | NA | NA | NA | Current anxiety disorder diagnosis or treatment with anxiolytic medication | Anxiety disorder was defined as the use of anxiolytic medication gathered by using continuous data collection with linkage of pharmacy records |
| Use of Aspirin | NA | NA | NA | Participants were identified as exposed to medications if they had received at least two consecutive prescriptions in the 12 months before baseline | Information on aspirin use was gathered by using continuous data collection with linkage of pharmacy records |
| Use of non-steroidal anti-inflammatory drugs (NSAIDs) | NA | NA | NA | Participants were identified as exposed to medications if they had received at least two consecutive prescriptions in the 12 months before baseline (NSAIDs, excluding aspirin) | Information on use of non-steroidal anti-inflammatory drugs was gathered by using continuous data collection with linkage of pharmacy records(7) |
| Cognitively stimulating activities | NA | NA | A structured interview focused on cognitive activities in late life (reading books and newspapers, playing games + social economic status where participant grew up) | NA | NA |
| Pesticide exposure | NA | NA | Dichotomized in never (reference) and ever (Questions specifically developed for ANU-ADRI) | NA | NA |
| Calendar year | NA | NA | NA | Calendar year at baseline to account for temporal trends | Calendar year at baseline to account for temporal trends |

**Abbreviations:** NA=not applicable, PCR-RFLP=Polymerase Chain Reaction-Restriction fragment length polymorphism, NHMRC=National Health and Medical Research Council, IPAQ=International Physical Activity Questionnaires, MET=metabolic equivalent of task, CES-D=Center for Epidemiologic Studies Depression Scale, TBI=traumatic brain injury, FFQ=food frequency questionnaire, TIA=transient ischemic attack and, NSAID=non-steroidal anti-inflammatory drugs.

**Table 2.** Distributions of the included predictors in the included models and validation sample

| **Prediction model** | **CAIDE** | **BDSI** | | | | **ANU-ADRI** | | | **DRS** | | **Validation study** |
| --- | --- | --- | --- | --- | --- | --- | --- | --- | --- | --- | --- |
| **Study population** | **CAIDE** | **CHS** | **FHS** | **HRS** | **SALSA** | **MAP** | **KP** | **CHS** | **THIN, 60-79 years** | **THIN, 80-95 years** | **Rotterdam Study** |
| **Study characteristics** | | | | | | | | | | | |
| Study type | Population based cohort study | Population based cohort study | Population based cohort study | Population based cohort study | Population based cohort study | Population based cohort study | Population based cohort study | Population based cohort study | Data derived from routine clinical practice | Data derived from routine clinical practice | Population based cohort study |
| Sample size, number | 1,409 | 2,794 | 2,411 | 13,889 | 1,125 | 1,164 | 1,301 | 3,375 | 800,013 | 130,382 | 6,667 |
| Geographical location | Finland | United States | United States | United States | United States | United States | Sweden | United States | United Kingdom | United Kingdom | The Netherlands |
| **Participant characteristics** | | | | | | | | | | | |
| Age at baseline [range] | 50.3 (6.0)  [39-64] | 72.9 (3.2)  [65-79] | 72.1 (4.4)  [65-79] | 71.3 (4.2)  [65-79] | 71.3 (4.0)  [65-79] | 79.8 (7.4)  [54-100] | 81.5 (5.0)  [74-100] | 72.3 (4.9)  [62-95] | 65.6 (6.08)  [60-79] | 84.8 (3.93)  [80-95] | 69.1 (8.2)  [55-105] |
| Sex, female | 875 (62.1) | 1,665 (59.6) | 1,338 (55.5) | 7,734 (56.5) | 643 (57.2) | 864 (75.2) | 976 (75.0) | 1,994 (59.1) | 413,974 (51.8) | 86,096 (66.0) | 3,787 (56.8) |
| Body mass index | 26.6 (3.7) |  |  |  |  | NA | NA | NA | 28.5 (5.44) | 25.7 (4.65) | 27.0 (4.0) |
| <18.5 | NA | 38 (1.4) | 22 (0.9) | 189 (1.4) | 0 (0) | NA | NA | NA | NA | NA | 49 (0.7) |
| 18.5-24.9 | NA | 940 (34.2) | 728 (30.2) | 4,752 (35.3) | 226 (20.1) | NA | NA | NA | NA | NA | 2,011 (30.2) |
| 25.0-29.9 | NA | 1,198 (43.6) | 1,030 (42.7) | 5,665 (40.5) | 423 (37.6) | NA | NA | NA | NA | NA | 3,053 (45.8) |
| ≥30 | NA | 573 (20.8) | 631 (26.2) | 3,277 (22.7) | 476 (42.3) | NA | NA | NA | NA | NA | 1,316 (19.7) |
| Systolic blood pressure | 144 (19.7) | NA | NA | NA | NA | NA | NA | NA | 142.4 (17.0) | 146.9 (19.8) | 143.5 (21.4) |
| Hypertension | NA | 1,754 (63.1) | 1,531 (63.7) | 7,678 (54.7) | 802 (71.3) | NA | NA | NA | NA | NA | 3,467 (52.0) |
| Education, years | 8.6 (3.4) |  |  |  |  | NA | NA | NA | NA | NA | 11.4 (3.6) |
| <6 | NA | NA | NA | NA | NA | NA | NA | NA | NA | NA | 0 (0) |
| <8 | NA | NA | NA | NA | NA | 42 (3.5) | 654 (50.3) | 367 (11.0) | NA | NA | 1,393 (20.9) |
| 7-9 | NA | NA | NA | NA | NA | NA | NA | NA | NA | NA | 1,393 (20.9) |
| 8-11 | NA | NA | NA | NA | NA | 60 (5.0) | 253 (19.4) | 439 (13.1) | NA | NA | 2,327 (34.9) |
| >9 | NA | NA | NA | NA | NA | NA | NA | NA | NA | NA | 5,154 (77.3) |
| >11 | NA | NA | NA | NA | NA | 1,061 (87.8) | 389 (29.9) | 2,537 (75.9) | NA | NA | 2,827 (42.4) |
| <12 | NA | 648 (23.2) | 322 (13.4) | 3,844 (25.8) | 803 (71.4) | NA | NA | NA | NA | NA | 3,720 (55.8) |
| Alcohol consumption | NA | NA | NA | NA | NA | 967 (80.9) | 577 (44.4) | 1,753 (52.7) | 22,308 (2.8) † | 921 (0.7) † | 5,477 (8.2) |
| Smoking |  | NA | NA | NA | NA |  |  |  |  |  |  |
| Never | NA | NA | NA | NA | NA | 685 (56.7) | 867 (66.7) | 1,558 (46.5) | 323,345 (42.8) | 63,684 (56.2) | 2,059 (30.9) |
| Former | NA | NA | NA | NA | NA | 432 (35.8) | NA | 1,426 (42.6) | 286,763 (37.9) | 39,778 (35.1) | 3,222 (48.3) |
| Current | 605 (42.9) | NA | NA | NA | NA | 44 (3.6) | 104 (8.0) | 364 (10.9) | 146,007 (19.3) | 9,929 (8.8) | 1,323 (19.8) |
| Total cholesterol, mmol/L | 6.72 (1.20) | NA | NA | NA | NA | NA | NA | NA | 5.21 (1.13) | 4.99 (1.19) | 5.80 (0.98) |
| Hypercholesterolemia | NA | NA | NA | NA | NA | NA | NA | NA | NA | NA | 1,479 (22.2) |
| High-density lipoprotein cholesterol, mmol/L | NA | NA | NA | NA | NA | NA | NA | NA | 1.42 (0.41) | 1.50 (0.44) | 1.39 (0.39) |
| Total-to-HDL-cholesterol ratio | NA | NA | NA | NA | NA | NA | NA | NA | 3.38 (1.17) | 3.49 (1.10) | 4.47 (1.32) |
| Physical activity |  | NA | NA | NA | NA |  |  |  | NA | NA |  |
| Low | 812 (57.6) ‡ | NA | NA | NA | NA | 465 (38.5) | NA | 993 (29.7) | NA | NA | 3,766 (56.5) |
| Middle | NA | NA | NA | NA | NA | 495 (41.0) | NA | 1,677 (50.2) | NA | NA | 1,408 (21.1) |
| High | NA | NA | NA | NA | NA | 203 (16.8) | NA | 671 (20.1) | NA | NA | 1,260 (18.9) |
| History of diabetes | NA | 289 (10.4) | 341 (14.1) | 2,465 (16.9) | 373 (33.2) | 147 (2.2) | 114 (8.5) | 537 (16.0) | 70,377 (8.8) | 12,762 (9.8) | 717 (10.8) |
| History of stroke | NA | 125 (4.6) | 60 (2.5) | 946 (6.8) | 108 (9.6) | NA | NA | NA | 38,976 (4.9) § | 20,221 (15.5) § | 244 (3.7) |
| History of TIA | NA | NA | NA | NA | NA | NA | NA | NA |  |  | 342 (5.1) |
| History of traumatic brain injury | NA | NA | NA | NA | NA | 65 (5.4) | 86 (6.6) | NA | NA | NA | 442 (6.6) |
| History of atrial fibrillation | NA | NA | NA | NA | NA | NA | NA | NA | 24,763 (3.1) | 14,518 (11.1) | 335 (5.0) |
| Depressive symptoms | NA | 499 (17.9) | 225 (11.9) | 3,974 (27.7) | 285 (24.4) | NA | NA | 180 (5.3) | 83,464 (10.4) | 17,201 (13.2) | 508 (7.6) |
| Social engagement or local deprivation¶ | NA | NA | NA | NA | NA |  |  | NA |  |  |  |
| Low | NA | NA | NA | NA | NA | 125 (10.3) | 84 (6.5) | NA | 134,103 (16.8) | 26,758 (20.5) | 1,130 (16.9) |
| Medium-Low | NA | NA | NA | NA | NA | 422 (34.9) | 880 (67.6) | NA | 166,956 (20.9) | 28,970 (22.2) | 4,827 (72.4) |
| Medium-High | NA | NA | NA | NA | NA | 328 (27.2) | 226 (17.4) | NA | 194,637 (24.3) | 30,143 (23.1) | 661 (9.9) |
| High | NA | NA | NA | NA | NA | 97 (8.0) | 13 (1.0) | NA | 218,198 (27.3) | 26,643 (20.4) | 19 (0.3) |
| Fish intake (serves/week) | NA | NA | NA | NA | NA | NA | NA |  | NA | NA |  |
| 0 – 0.25 | NA | NA | NA | NA | NA | NA | NA | 388 (13.2) | NA | NA | 15 (0.2) |
| 0.26 – 2.0 | NA | NA | NA | NA | NA | NA | NA | 1,168 (39.8) | NA | NA | 88 (1.3) |
| 2.1 – 4.0 | NA | NA | NA | NA | NA | NA | NA | 1,263 (43.0) | NA | NA | 1,837 (27.6) |
| ≥4.0 | NA | NA | NA | NA | NA | NA | NA | 119 (4.1) | NA | NA | 1,529 (22.9) |
| Need help with finances or medications | NA | 16 (0.6) | 41 (1.7) | 305 (2.2) | 122 (10.8) | NA | NA | NA | NA | NA | 1,180 (17.7) |
| Use of antihypertensive drugs | NA | NA | NA | NA | NA | NA | NA | NA | 274,657 (34.3) | 58,323 (44.7) | 1,551 (23.3) |
| Anxiety or use of anxiolytics | NA | NA | NA | NA | NA | NA | NA | NA | 29,690 (3.7) | 5,953 (4.6) | 790 (11.8) |
| Use of aspirin | NA | NA | NA | NA | NA | NA | NA | NA | 127,550 (15.9) | 41,448 (31.8) | 1,158 (17.4) |
| Use of NSAIDs (excluding aspirin) | NA | NA | NA | NA | NA | NA | NA | NA | 98,397 (12.3) | 15,056 (11.6) | 569 (8.5) |
| Cognitively stimulating activities | NA | NA | NA | NA | NA | NA | NA | NA | NA | NA | NA |
| Low | NA | NA | NA | NA | NA | 408 (33.8) | NA | NA | NA | NA | NA |
| Moderate | NA | NA | NA | NA | NA | 600 (49.7) | NA | NA | NA | NA | NA |
| High | NA | NA | NA | NA | NA | 155 (12.8) | NA | NA | NA | NA | NA |

Data are shown for non-imputed data. Values are counts (percentages) or means (standard deviation).

*Obesity was defined as a body mass index of >30 kg/m^2^.

†Heavy alcohol drinking (more than 56 units per week for men/49 units per week for women).

‡Physically inactive: participants having leisure-time physical activity less often than twice per week.

§Participants with a history of stroke or transient ischemic attack.

¶ Used as a social deprivation index for the Dementia Risk Score.

**Abbreviations:** CAIDE=Cardiovascular Risk factors, Aging, and Dementia study, BDSI=Brief Dementia Screening Indicator, ANU-ADRI=Australian National University Alzheimer’s Disease Risk Index, DRS=Dementia Risk Score, CHS=Cardiovascular Health Study, FHS=Framingham Heart

Study, HRS=Health and Retirement Study, SALSA=Sacramento Area Latino Study on Aging, MAP=Rush Memory and Aging Study, KP=Kungsholmen Project,

THIN=The Health Improvement Network, NA=not applicable, TBI=traumatic brain injury, TIA=transient ischemic attack and, NSAID=non-steroidal anti-inflammatory

drugs.

**Descriptions of the predictors in the validation sample**

*Demographics*

Age and gender were derived from when participants entered the Rotterdam Study. The variable education was derived from self-reported history harmonized in years of education according to the UNESCO classification (8).

*Hypertension*

Systolic and diastolic blood pressures were assessed at the right arm and the mean of two measurements was used in the analyses. Hypertension was defined according to definitions of the included models.

*Hypercholesterolemia*

Total cholesterol, high-density lipoprotein (HDL) cholesterol and triglyceride concentrations were measured from serum or plasma extracted from whole blood, using an automated enzymatic procedure (Boehringer Mannheim System) (9). Hypercholesterolemia was defined in correspondence with the used cut-off of the included models.

*Obesity*

Body mass index (BMI) was calculated as weight in kilograms divided by length in meters squared (kg/m^2^).

*Physical inactivity*

Physical activity levels were assessed using a validated adapted version of Zutphen Physical Activity Questionnaire (10) and expressed in Metabolic Equivalent of Task hours per week (METhours/week) (11). The METhours/week are the MET-values of specific activities (walking, cycling, domestic work, sports and gardening) with time in hours per week spent in that activity. Categories were calculated based on International Physical Activity Questionnaire (IPAQ) (12) and also expressed in METhours/week.

*Type 2 diabetes*

Type 2 diabetes was defined as fasting serum glucose levels ≥7.0 mmol/L, non-fasting serum glucose levels ≥11.0 mmol/L (if fasting samples were not available), or the use of blood glucose-lowering therapy (7).

*Depressive symptoms*

A structured interview to screen for depressive symptoms was performed and a semi-structured interview to diagnose disorder. Participants were screened with the Center for Epidemiologic Studies Depression (CES-D) Scale during the home interview (13). For the BDSI model we used both the CES-D scale and use of antidepressants. Depression was defined as a CES-D score of >16.

*Head trauma*

A home interview to screen for serious head trauma or concussion was performed at baseline and during follow-up using the following questions: “Did you ever have a serious head trauma or a concussion?” and “Were you ever unconscious because of a head trauma?”

*Smoking*

Study participants were enquired about their smoking habits during each home interview. According to their smoking history, participants are classified as never smokers, former or current smokers.

*Cognitive activity*

Cognitive activity has not been assessed systematically in the Rotterdam Study, and therefore could not be included in our analyses.

*Social network and engagement*

Social and network engagement was constructed using three domains based on various questionnaires. We included the marital status, living arrangements (living alone, with spouse of with others) and asking if the participant felt lonely during the past week using the CES-D questionnaire. The same domains were used to emulate the social deprivation variable.

*Alcohol*

During the structured home interview, participants were asked the number of drinks per week.

*Fish servings per week*

Participants were specifically asked to indicate the frequency, amount, and kind of fish they had eaten using a validated Food Frequency Questionnaire (FFQ). Portions were calculated in grams per day (14).

*Pesticide exposure*

Pesticide exposure has not been evaluated in the Rotterdam Study, therefore we could not include this in our analyses.

*Stroke and transient ischemic attack (TIA)*

At baseline, history of stroke or TIA was assessed by interview and verified using medical records. Study participants were continuously followed up for occurrence of incident stroke, based on information collected continuously from medical records of general practitioners and neurologists. Nursing home physicians’ medical records and general practitioners’ medical records of participants who moved out of the study area were checked on a regular basis as well.

Information from GPs and hospital records was collected from participants with a potential stroke or TIA. Research physicians reviewed the information and an experienced vascular neurologist verified the diagnoses according to World Health Organization criteria (15).

*Needs help with finances or medications*

Study participants were enquired about their financial management and about medication use with a questionnaire about activities of daily living.

*Anxiety*

Study participants were dichotomized having a current anxiety disorder or not based on the use of anxiolytic medication (Anatomical Therapeutic Chemical Classification codes N05B).

*History of atrial fibrillation (AF)*

Prevalent AF was assessed using 3 methods. Electrocardiography (ECG) was performed at baseline and at each follow-up examination. The ECGs were analyzed by the Modular ECG Analysis System (MEANS) to verify the diagnosis of AF. Every ECG with any rhythm disorder identified by the MEANS program was coded independently by 2 research physicians who were masked to the MEANS diagnosis. In case of disagreement between the research physicians, a cardiologist decided on the final diagnosis. Second, information for all participants concerning the presence of AF and the date of onset was obtained from general practitioner records and medical specialists. Third, information on all hospital discharges from a nationwide medical registry (Landelijke Medische Registratie) was collected. We did not differentiate between AF and atrial flutter when identifying cases since these conditions are very similar with regard to risk factors and consequences (16).

*Use of aspirin, NSAIDs and anxiolytic medication*

On each of the study rounds study participants were enquired about the use of medicinal agents, vitamins, food supplements and 'over-the-counter' drugs. Furthermore, drug exposure has been monitored continuously for nearly all participants through digital linkage with computerized records of the pharmacies in the study district.

## Appendix C.

## Full model equations for the risk of dementia as applied in the validation cohort

1. Cardiovascular Risk Factors, Aging, and Dementia (CAIDE) risk score equation:

P(dementia)= (^exp^ ^(-7.406 (intercept) + 0.796×(follow-up time) + 0.401×(risk score)^)

1+(^exp(-7.406 (intercept) + 0.796 × (follow-up time)+ 0.401× (risk score)^)

2. Dementia Risk Score (DRS) risk score equation:

Formula aged 60-79 at baseline:

P(dementia) =

0.20921×(age - 65.608) + -0.00339×(age - 65.608)×(age - 65.608) + -0.0616×(BMI - 27.501) + 0.002508×(BMI - 27.501)×(BMI - 27.501) + 0.12854×1(if female) + -0.13199×1(if anti-hypertensive use) + 0.04477×(calendar year - 2003.719) + 0.013371×1(if Townsend score 2) + 0.117904×1(if Townsend score 3) + 0.201776×1(if Townsend score 4) + 0.225529×1 (if Townsend score 5) + -0.06792×1(if ex-smoker) + -0.08657×1(if current smoker) + 0.443535×(if heavy drinker) + 0.833612×1(if depressed) + 0.252833×1(aspirin use) + 0.577207×1(if prevalent stroke or TIA) + 0.220728×1(if prevalent atrial fibrillation)+ 0.286701×1(if prevalent type 2 diabetes).

Define S = 0.9969.

Then predicted 5-year risk (as a percentage) = 100×[1-S^exp(P)^].

Formula aged 80-95 at baseline:

P(dementia) =

0.055×(age – 84.8) + -0.005×(age – 84.8)×(age – 84.8) + -0.05×(BMI – 25.7) + 0.160×1(if female) + -0.249×1(if hypertensive use) + - 0.006×(systolic blood pressure – 146.9) + 0.042×(lipid ratio – 3.49) + 0.074×(calendar year - 2003.719) + -0.178×1(if ex-smoker) + -0.134×1(if current smoker) + 0.256×(if heavy drinker) + 0.400×1(if depressed) + 0.092×1(if aspirin use) + 0.242×1(if prevalent stroke or TIA) + 0.057×1(if prevalent atrial fibrillation)+ 0.183×1(if prevalent type 2 diabetes) + 0.136 × (if anxiolytic drug use) + -0.157 × (if NSAID use).

Define S = 0.9277.

Then predicted 5-year risk (as a percentage) = 100×[1-S^exp(P)^].

## Appendix D.

## Supplementary tables

## Table 1. Reported performance of dementia prediction models

| Prediction model | C-statistics (95% CI)  in data the model was developed on | Validation in literature using external data | Predicted time horizon, years |
| --- | --- | --- | --- |
| CAIDE | 0.77 (0.71-0.83) | 0.49 to 0.75 | 20 |
| CAIDE + *APOE*-ε4 carrier | 0.79 (0.74-0.85) | NA | 20 |
| BDSI | 0.68 to 0.78 | 0.68 to 0.78 | 6 |
| ANU-ADRI | NA† | 0.64 to 0.74 | 6 |
| DRS |  |  |  |
| Aged 60-79 years | NA† | 0.84 (0.81-0.87) | 5 |
| Aged 80-95 years | NA† | 0.56 (0.55-0.58) | 5 |

* No confidence interval published.

† No performance measures reported for the development sample.

Abbreviations: CI=confidence interval, NA=not applicable, *APOE=*apolipoprotein E, CAIDE=Cardiovascular Risk

factors, Aging, and Dementia study, *APOE*=apolipoprotein E, BDSI=Brief Dementia Screening Indicator, ANU-

ADRI=Australian National University Alzheimer’s Disease Risk Index and, DRS=Dementia Risk Score.

### Table 2. Discriminative ability for all-cause dementia per prediction model in the Rotterdam Study while restricted to the specific age range for which the models were designed

| Prediction model | C-statistics at various follow-up horizons (95% CI) | | | |
| --- | --- | --- | --- | --- |
|  | **2 years** | **5 years** | **10 years** | **15 years** |
| Participants aged 65-79 years | **n/N=33/3,481** | **n/N=133/3,481** | **n/N=315/3,481** | **n/N=578/3,481** |
| BDSI | 0.72 (0.62-0.82) | 0.69 (0.64-0.73) | 0.67 (0.64-0.70) | 0.64 (0.61-0.66) |
| Age only | 0.71 (0.61-0.81) | 0.69 (0.64-0.74) | 0.68 (0.65-0.71) | 0.66 (0.63-0.68) |
|  |  |  |  |  |
| Participants aged 60-79 years | **n/N=34/5,019** | **n/N=141/5,019** | **n/N=345/5,019** | **n/N=644/5,019** |
| DRS | 0.80 (0.70-0.89) | 0.77 (0.72-0.81) | 0.75 (0.72-0.78) | 0.73 (0.71-0.75) |
| Age only | 0.78 (0.68-0.88) | 0.75 (0.71-0.80) | 0.74 (0.71-0.77) | 0.73 (0.70-0.75) |
|  |  |  |  |  |
| Participants aged 80-95 years | **n/N=29/709** | **n/N=91/709** | **n/N=167/709** | **n/N=192/709** |
| DRS | 0.60 (0.49-0.71) | 0.56 (0.50-62) | 0.56 (0.51-0.61) | 0.56 (0.51-0.60) |
| Age only | 0.53 (0.42-0.64) | 0.57 (0.51-0.63) | 0.56 (0.51-0.60) | 0.55 (0.51-0.60) |

Abbreviations: CI=confidence interval, n=number of cases, N=number of people at risk, BDSI=Brief Dementia Screening

Indicator and, DRS=Dementia Risk Score.

**Table 3.** Discriminative ability for Alzheimer’s disease per prediction model in the Rotterdam Study

| Prediction model | C-statistics at various follow-up horizons (95% CI) | | | |
| --- | --- | --- | --- | --- |
|  | **2 years**  **n/N=58/6,667** | **5 years**  **n/N=197/6,667** | **10 years**  **n/N=421/6,667** | **15 years**  **n/N=682/6,667** |
| CAIDE | 0.51 (0.43-0.59) | 0.54 (0.50-0.58) | 0.55 (0.53-0.58) | 0.55 (0.53-0.57) |
| Age only | NA | NA | NA | NA |
|  |  |  |  |  |
| BDSI | 0.83 (0.75-0.90) | 0.81 (0.76-0.85) | 0.80 (0.77-0.83) | 0.78 (0.75-0.80) |
| Age only | 0.83 (0.76-0.91) | 0.82 (0.78-0.86) | 0.82 (0.79-0.84) | 0.80 (0.78-0.83) |
|  |  |  |  |  |
| ANU-ADRI | 0.80 (0.75-0.85) | 0.78 (0.75-0.80) | 0.75 (0.73-0.77) | 0.69 (0.68-0.71) |
| Age only | 0.83 (0.78-0.87) | 0.80 (0.78-0.83) | 0.78 (0.76-0.80) | 0.74 (0.72-0.75) |
|  |  |  |  |  |
| DRS | 0.84 (0.77-0.92) | 0.82 (0.78-0.86) | 0.82 (0.79-0.84) | 0.80 (0.78-0.82) |
| Age only | 0.83 (0.76-0.91) | 0.82 (0.78-0.86) | 0.82 (0.79-0.84) | 0.80 (0.78-0.83) |

Abbreviations: CI=confidence interval, n=number of cases, N=number of people at risk, CAIDE=Cardiovascular Risk factors, Aging and, Dementia study, NA=not applicable, BDSI=Brief Dementia Screening Indicator, ANU-ADRI=Australian National University Alzheimer’s Disease Risk Index and, DRS=Dementia Risk Score.

### Table 4. Discriminative ability for all-cause dementia prediction models in the Rotterdam Study, excluding the first four years of follow-up

| Prediction model | C-statistics (95% CI) | |
| --- | --- | --- |
|  | **10 years**  **n/N=368/6,035** | **15 years**  **n/N=700/6,035** |
| CAIDE | 0.56 (0.53-0.59) | 0.55 (0.53-0.58) |
| Age only | NA | NA |
|  |  |  |
| BDSI | 0.75 (0.72-0.79) | 0.72 (0.70-0.74) |
| Age only | 0.79 (0.77-0.82) | 0.78 (0.76-0.80) |
|  |  |  |
| ANU-ADRI | 0.75 (0.73-0.78) | 0.70 (0.68-0.72) |
| Age only | 0.77 (0.75-0.79) | 0.73 (0.71-0.74) |
|  |  |  |
| DRS | 0.80 (0.77-0.83) | 0.78 (0.76-0.80) |
| Age only | 0.80 (0.77-0.83) | 0.78 (0.76-0.80) |

Abbreviations: CI=confidence interval, n=number of cases, N=number of people at risk, CAIDE=Cardiovascular Risk factors, Aging and, Dementia study, NA=not applicable, BDSI=Brief Dementia Screening Indicator, ANU-ADRI=Australian National University Alzheimer’s Disease Risk Index and, DRS=Dementia Risk Score.

### Table 5. Discriminative performance for all-cause dementia prediction models in the Rotterdam Study, stratified by age at baseline

| Prediction model | C-statistics at various follow-up horizons (95% CI) | | | |
| --- | --- | --- | --- | --- |
|  | **2 years**  **n/N=34/5,947** | **5 years**  **n/N=142/5,947** | **10 years**  **n/N=347/5,947** | **15 years**  **n/N=654/5,947** |
| Participants aged <80 years |  |  |  |  |
| CAIDE | 0.49 (0.40-0.59) | 0.55 (0.50-0.60) | 0.56 (0.53-0.59) | 0.55 (0.53-0.57) |
| Age only | NA | NA | NA | NA |
|  |  |  |  |  |
| BDSI | 0.80 (0.70-0.90) | 0.76 (0.71-0.81) | 0.73 (0.70-0.76) | 0.71 (0.68-0.73) |
| Age only | 0.81 (0.72-0.91) | 0.79 (0.74-0.84) | 0.78 (0.75-0.81) | 0.77 (0.74-0.79) |
|  |  |  |  |  |
| ANU-ADRI | 0.77 (0.70-0.84) | 0.74 (0.70-0.75) | 0.72 (0.70-0.75) | 0.69 (0.67-0.71) |
| Age only | 0.76 (0.68-0.83) | 0.74 (0.70-0.78) | 0.75 (0.73-0.77) | 0.72 (0.70-0.74) |
|  |  |  |  |  |
| DRS | 0.83 (0.73-0.92) | 0.80 (0.75-0.85) | 0.79 (0.76-0.82) | 0.77 (0.75-0.79) |
| Age only | 0.81 (0.72-0.91) | 0.79 (0.74-0.84) | 0.78 (0.75-0.81) | 0.77 (0.75-0.79) |
| Participants aged ≥80 years | **n/N=29/720** | **n/N=91/720** | **n/N=168/720** | **n/N=193/720** |
| CAIDE | 0.54 (0.44-0.64) | 0.51 (0.45-0.58) | 0.50 (0.45-0.55) | 0.52 (0.47-0.56) |
| Age only | NA | NA | NA | NA |
|  |  |  |  |  |
| BDSI | 0.52 (0.41-0.63) | 0.56 (0.50-0.62) | 0.55 (0.51-0.60) | 0.55 (0.51-0.59) |
| Age only | 0.53 (0.42-0.64) | 0.56 (0.50-0.62) | 0.56 (0.51-0.60) | 0.55 (0.51-0.60) |
|  |  |  |  |  |
| ANU-ADRI | 0.62 (0.51-0.72) | 0.50 (0.43-0.56) | 0.53 (0.48-0.58) | 0.54 (0.49-0.58) |
| Age only | 0.54 (0.44-0.64) | 0.53 (0.47-0.59) | 0.52 (0.48-0.56) | 0.52 (0.49-0.56) |
|  |  |  |  |  |
| DRS | 0.56 (0.45-0.67) | 0.56 (0.50-0.62) | 0.56 (0.51-0.60) | 0.56 (0.51-0.60) |
| Age only | 0.53 (0.42-0.64) | 0.57 (0.51-0.63) | 0.56 (0.51-0.60) | 0.55 (0.51-0.60) |

Abbreviations: CI=confidence interval, n=number of cases, N=number of people at risk, CAIDE=Cardiovascular Risk factors, Aging, and Dementia study, NA=not applicable, BDSI=Brief Dementia Screening Indicator, ANU-ADRI=Australian National University Alzheimer’s Disease Risk Index and, DRS=Dementia Risk Score.

| **Section/Topic** | **Item** | **Checklist Item** | **Page** |
| --- | --- | --- | --- |
| **Title and abstract** | | | |
| Title | 1 | Identify the study as developing and/or validating a multivariable prediction model, the target population, and the outcome to be predicted. | 1 |
| Abstract | 2 | Provide a summary of objectives, study design, setting, participants, sample size, predictors, outcome, statistical analysis, results, and conclusions. | 4 |
| **Introduction** | | | |
| Background and objectives | 3a | Explain the medical context (including whether diagnostic or prognostic) and rationale for developing or validating the multivariable prediction model, including references to existing models. | 6 |
|  | 3b | Specify the objectives, including whether the study describes the development or validation of the model or both. | 7 |
| **Methods** | | | |
| Source of data | 4a | Describe the study design or source of data (e.g., randomized trial, cohort, or registry data), separately for the development and validation data sets, if applicable. | 9,10 |
|  | 4b | Specify the key study dates, including start of accrual; end of accrual; and, if applicable, end of follow-up. | 10,11 |
| Participants | 5a | Specify key elements of the study setting (e.g., primary care, secondary care, general population) including number and location of centres. | 10,11 |
|  | 5b | Describe eligibility criteria for participants. | 10,11 |
|  | 5c | Give details of treatments received, if relevant. | NA |
| Outcome | 6a | Clearly define the outcome that is predicted by the prediction model, including how and when assessed. | 9-10 |
|  | 6b | Report any actions to blind assessment of the outcome to be predicted. | 10 |
| Predictors | 7a | Clearly define all predictors used in developing or validating the multivariable prediction model, including how and when they were measured. | 10 |
|  | 7b | Report any actions to blind assessment of predictors for the outcome and other predictors. | 10 |
| Sample size | 8 | Explain how the study size was arrived at. | 9 |
| Missing data | 9 | Describe how missing data were handled (e.g., complete-case analysis, single imputation, multiple imputation) with details of any imputation method. | 13 |
| Statistical analysis methods | 10c | For validation, describe how the predictions were calculated. | 11-12 |
|  | 10d | Specify all measures used to assess model performance and, if relevant, to compare multiple models. | 11-12 |
|  | 10e | Describe any model updating (e.g., recalibration) arising from the validation, if done. | 12 |
| Risk groups | 11 | Provide details on how risk groups were created, if done. | NA |
| Development vs. validation | 12 | For validation, identify any differences from the development data in setting, eligibility criteria, outcome, and predictors. | 9,10,  11 |
| **Results** | | | |
| Participants | 13a | Describe the flow of participants through the study, including the number of participants with and without the outcome and, if applicable, a summary of the follow-up time. A diagram may be helpful. | 9 |
|  | 13b | Describe the characteristics of the participants (basic demographics, clinical features, available predictors), including the number of participants with missing data for predictors and outcome. | 13,23 |
|  | 13c | For validation, show a comparison with the development data of the distribution of important variables (demographics, predictors and outcome). | 10,13 |
| Model performance | 16 | Report performance measures (with CIs) for the prediction model. | 13,14,25 |
| Model-updating | 17 | If done, report the results from any model updating (i.e., model specification, model performance). | 14,15,26,27 |
| **Discussion** | | | |
| Limitations | 18 | Discuss any limitations of the study (such as nonrepresentative sample, few events per predictor, missing data). | 17,18 |
| Interpretation | 19a | For validation, discuss the results with reference to performance in the development data, and any other validation data. | 17,18 |
|  | 19b | Give an overall interpretation of the results, considering objectives, limitations, results from similar studies, and other relevant evidence. | 15,16 |
| Implications | 20 | Discuss the potential clinical use of the model and implications for future research. | 16,17,18 |
| **Other information** | | | |
| Supplementary information | 21 | Provide information about the availability of supplementary resources, such as study protocol, Web calculator, and data sets. | 8-10,13 |
| Funding | 22 | Give the source of funding and the role of the funders for the present study. | 20 |

**Appendix E.** TRIPOD Checklist: Prediction Model Validation

## Supplementary references

1. Kivipelto M, Ngandu T, Laatikainen T, Winblad B, Soininen H, Tuomilehto J. Risk score for the prediction of dementia risk in 20 years among middle aged people: a longitudinal, population-based study. Lancet Neurol. 2006;5(9):735-41.

2. Exalto LG, Quesenberry CP, Barnes D, Kivipelto M, Biessels GJ, Whitmer RA. Midlife risk score for the prediction of dementia four decades later. Alzheimers Dement. 2014;10(5):562-70.

3. Barnes DE, Beiser AS, Lee A, Langa KM, Koyama A, Preis SR, et al. Development and validation of a brief dementia screening indicator for primary care. Alzheimers Dement. 2014;10(6):656-65 e1.

4. Anstey KJ, Cherbuin N, Herath PM. Development of a new method for assessing global risk of Alzheimer's disease for use in population health approaches to prevention. Prev Sci. 2013;14(4):411-21.

5. Anstey KJ, Cherbuin N, Herath PM, Qiu C, Kuller LH, Lopez OL, et al. A self-report risk index to predict occurrence of dementia in three independent cohorts of older adults: the ANU-ADRI. PLoS One. 2014;9(1):e86141.

6. Walters K, Hardoon S, Petersen I, Iliffe S, Omar RZ, Nazareth I, et al. Predicting dementia risk in primary care: development and validation of the Dementia Risk Score using routinely collected data. BMC Med. 2016;14:6.

7. Leening MJ, Kavousi M, Heeringa J, van Rooij FJ, Verkroost-van Heemst J, Deckers JW, et al. Methods of data collection and definitions of cardiac outcomes in the Rotterdam Study. Eur J Epidemiol. 2012;27(3):173-85.

8. United Nations Educational SaCOU. International Standard Classification of Education (ISCED) 1976. Available from: <http://unesdoc.unesco.org/images/0002/000209/020992eb.pdf>.

9. Postmus I, Deelen J, Sedaghat S, Trompet S, de Craen AJ, Heijmans BT, et al. LDL cholesterol still a problem in old age? A Mendelian randomization study. Int J Epidemiol. 2015;44(2):604-12.

10. Caspersen CJ, Bloemberg BP, Saris WH, Merritt RK, Kromhout D. The prevalence of selected physical activities and their relation with coronary heart disease risk factors in elderly men: the Zutphen Study, 1985. Am J Epidemiol. 1991;133(11):1078-92.

11. Koolhaas CM, Dhana K, Golubic R, Schoufour JD, Hofman A, van Rooij FJ, et al. Physical Activity Types and Coronary Heart Disease Risk in Middle-Aged and Elderly Persons: The Rotterdam Study. Am J Epidemiol. 2016;183(8):729-38.

12. Craig CL, Marshall AL, Sjostrom M, Bauman AE, Booth ML, Ainsworth BE, et al. International physical activity questionnaire: 12-country reliability and validity. Med Sci Sports Exerc. 2003;35(8):1381-95.

13. Beekman AT, Deeg DJ, Van Limbeek J, Braam AW, De Vries MZ, Van Tilburg W. Criterion validity of the Center for Epidemiologic Studies Depression scale (CES-D): results from a community-based sample of older subjects in The Netherlands. Psychol Med. 1997;27(1):231-5.

14. Heine-Broring RC, Brouwer IA, Proenca RV, van Rooij FJ, Hofman A, Oudkerk M, et al. Intake of fish and marine n-3 fatty acids in relation to coronary calcification: the Rotterdam Study. Am J Clin Nutr. 2010;91(5):1317-23.

15. Wieberdink RG, Ikram MA, Hofman A, Koudstaal PJ, Breteler MM. Trends in stroke incidence rates and stroke risk factors in Rotterdam, the Netherlands from 1990 to 2008. Eur J Epidemiol. 2012;27(4):287-95.

16. de Bruijn RF, Heeringa J, Wolters FJ, Franco OH, Stricker BH, Hofman A, et al. Association Between Atrial Fibrillation and Dementia in the General Population. JAMA Neurol. 2015;72(11):1288-94.
